# Complete metagenome-assembled genome sequence of *Solidesulfovibrio* sp. DCME from a dichloromethane dechlorinating microbial community

**DOI:** 10.1128/mra.00515-25

**Published:** 2025-06-30

**Authors:** Olivia Bulka, Elizabeth A. Edwards

**Affiliations:** 1 Department of Chemical Engineering and Applied Chemistry, University of Toronto 7938, Toronto, Canada; Montana State University, Bozeman, Montana, USA

**Keywords:** dehalogenation, dichloromethane, hydrogen, hydrogenase, bioremediation, cross-feeding, pangenome

## Abstract

Here, we announce the closed genome of *Solidesulfovibrio* sp. DCME, assembled from metagenomic sequencing of an anaerobic dichloromethane mineralizing enrichment culture. The *Solidesulfovibrio* genus is known to cycle hydrogen, a key process for facilitating dichloromethane mineralization, which nominates this microbe as an important player in its microbial community.

## ANNOUNCEMENT

We enriched an anaerobic dichloromethane (DCM)-degrading microbial community (called DCME) as a subculture from a chloroform- and DCM-dechlorinating culture that was originally derived from contaminated groundwater in California (SiREM, Guelph, ON) (1, 2). The DCME culture has been fed DCM as its sole electron donor and carbon source (~1 mM [aqueous] biweekly-monthly) since 2019 to enrich DCM-mineralizing microbes (1, 3). DCM mineralization relies on syntrophic hydrogenotrophs to maintain sufficiently low hydrogen partial pressures (4, 5). Here, we describe the assembly of a metagenome-assembled genome from a well-studied hydrogen cycling genus in the DCME community: *Solidesulfovibrio* sp. DCME.

DNA was extracted from 400 mL DCME using the Kingfisher Duo Prime MagMax Microbiome Kit (Thermo Scientific, Waltham, MA, USA), as detailed in our previous announcement (6). Samples were prepared for PacBio Sequel II sequencing by the Genome Quebec Innovation Centre (Montréal, QC, Canada) using the SMRTbell Express Template Prep Kit 2.0 (Pacific Biosciences, Menlo Park, CA, USA) without shearing or size selection. Canu v2.1.1 (7) was used to assemble 2,736,486 PacBio reads (*N*_*50*_ = 65,981); some parameters were adjusted from default for low-coverage metagenomes (genomeSize = 5m, maxInputCoverage = 10,000, corOutCoverage = 10,000, corMhapSensitivity = high, corMinCoverage = 0, redMemory = 32, oeaMemory = 32, batMemory = 200). Contigs longer than 2 Mb and flagged as circular by Canu were manually selected for further evaluation. Polypolish v0.60 (8, 9) was used to correct indels with 84,638,297 Illumina NovaSeq 6000 reads (150 bp) described in our previous announcement (10). Reads were mapped for visual inspection within Anvi’o v7 (11–13). Taxonomy was performed with GTDB-Tk v2.3.2 using reference data R207 (14, 15). The genome was compared to publicly available *Solidesulfovibrio* genomes in NCBI using the anvi-pan-genome workflow within Anvi’o v7 and IQ-TREE to construct a phylogenetic tree (Whelan and Goldman model, 1,000 bootstraps) (16). Hydrogenase sequences were aligned using clinker v0.0.27 (17).

**Fig 1:**
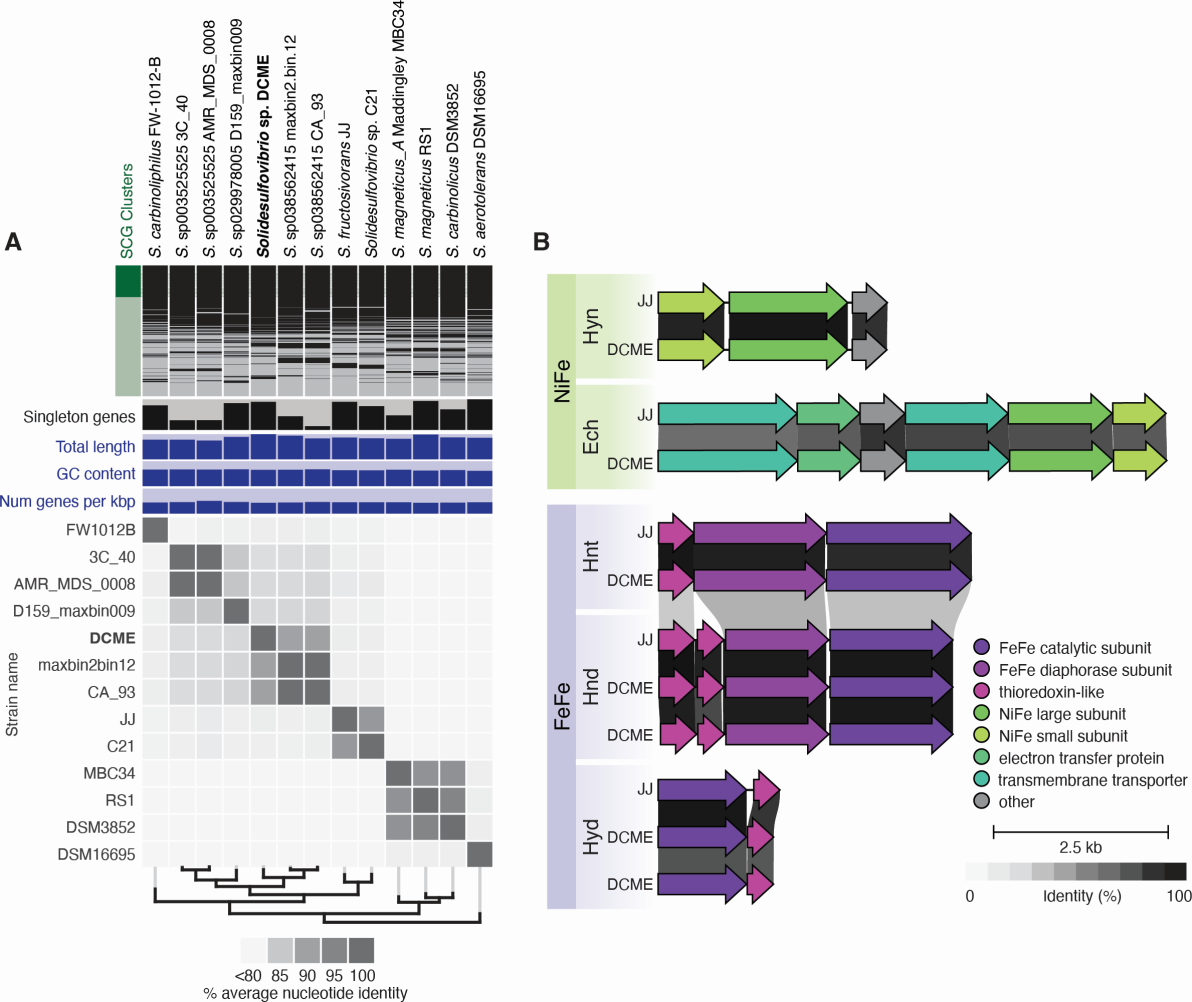
Comparison of the *Solidesulfovibrio* sp. DCME genome to other *Solidesulfovibrio* strains. (A) Pangenomic similarity to its genus in terms of homologous gene clusters, genome size and content, average nucleotide identity, and phylogenomic relation based on single-copy core genes (SGCs); (B) Hydrogenase gene clusters, compared with amino acid sequences from *Solidesulfovibrio fructosivorans* JJ (GCF_000179555).

The *Solidesulfovibrio* sp. DCME genome (5.39 Mb, 65.7% GC, mean read depth: 105.9×, 97.78% complete) was the only large circular sequence identified in this assembly. The overlap was identified by Canu and trimmed manually after visual inspection. The genome was oriented at the *dnaA* gene, using iRep to calculate GC skew (18). It was annotated using PGAP v6.1 (19), which predicted 4,653 genes, 4,697 coding sequences, 50 tRNAs, and two complete rRNA operons. Phylogenetically, strain DCME is nestled firmly in the *Solidesulfovibrio* genus but falls outside any known species (Fig. 1A). *Solidesulfovibrio* spp. have a complex relationship with hydrogen (20–22)—alternating production and consumption at different stages of metabolism—and strain DCME appears to be no exception. Its genome encodes seven hydrogenases (Fig. 1B), including two (NiFe) hydrogenases: an Ech-type (energy-conserving [23]) and a Hyn-type (periplasmic uptake [24, 25]), and five (FeFe) hydrogenases: two Hyd-type (periplasmic uptake [26]), two Hnd-type (reversible, electron bifurcating [27–30]), and one Hnt-type (trimeric, NAD[P]H-dependent [31, 32]) hydrogenase. The reliance of DCM mineralization on hydrogen consumption posits this species as an important syntrophic microbe in DCME, as seen in other communities (33, 34) and as discussed in our related work (5).

## Data Availability

All data are available in NCBI under BioProject PRJNA1013980. The *Solidesulfovibrio* sp. DCME genome is deposited under accession number CP188261. PacBio (SRR25941763) and Illumina (SRR27458441) reads are deposited in the Sequence Read Archive.
